# Understanding the role of nerves in head and neck cancers - a review

**DOI:** 10.3389/or.2024.1514004

**Published:** 2025-01-20

**Authors:** Krzysztof Rutkowski, Michał Gola, Janusz Godlewski, Anna Starzyńska, Giulia Marvaso, Federico Mastroleo, Maria Giulia Vincini, Alice Porazzi, Mattia Zaffaroni, Barbara Alicja Jereczek-Fossa

**Affiliations:** ^1^ Department of Hematology, Transplantology and Internal Medicine, Medical University of Warsaw, Warsaw, Poland; ^2^ Department of Human Histology and Embryology, Collegium Medicum, School of Medicine, University of Warmia and Mazury, Olsztyn, Poland; ^3^ Department of Oncology and Immuno-Oncology, Clinical Hospital of the Ministry of Internal Affairs and Administration with the Warmia-Mazury Oncology Centre, Olsztyn, Poland; ^4^ Department of Surgical Oncology, Clinical Hospital of the Ministry of Internal Affairs and Administration with the Warmia-Mazury Oncology Centre, Olsztyn, Poland; ^5^ Department of Oral Surgery, Medical University of Gdańsk, Gdańsk, Poland; ^6^ Department of Otolaryngology, Phoniatrics and Audiology, Collegium Medicum in Bydgoszcz, Nicolaus Copernicus University in Toruń, Bydgoszcz, Poland; ^7^ Division of Radiation Oncology, European Institute of Oncology (IEO), Instituto di Ricovero e Cura a Carattere Scientifico (IRCCS), Milan, Italy; ^8^ Department of Oncology and Hemato-Oncology, University of Milan, Milan, Italy

**Keywords:** head and neck cancer, squamous cell carcinoma, perineural invasion, tumor microenvironment, axonogenesis

## Abstract

Worldwide, head and neck cancers (HNCs) account for approximately 900,000 cases and 500,000 deaths annually, with their incidence continuing to rise. Carcinogenesis is a complex, multidimensional molecular process leading to cancer development, and in recent years, the role of nerves in the pathogenesis of various malignancies has been increasingly recognized. Thanks to the abundant innervation of the head and neck region, peripheral nervous system has gained considerable interest for its possible role in the development and progression of HNCs. Intratumoral parasympathetic, sympathetic, and sensory nerve fibers are emerging as key players and potential targets for novel anti-cancer and pain-relieving medications in different tumors, including HNCs. This review explores nerve-cancer interactions, including perineural invasion (PNI), cancer-related axonogenesis, neurogenesis, and nerve reprogramming, with an emphasis on their molecular mechanisms, mediators and clinical implications. PNI, an adverse histopathologic feature, has been widely investigated in HNCs. However, its prognostic value remains debated due to inconsistent results when classified dichotomously (present/absent). Emerging evidence suggests that quantitative and qualitative descriptions of PNI may better reflect its clinical usefulness. The review also examines therapies targeting nerve-cancer crosstalk and highlights the influence of HPV status on tumor innervation. By synthesizing current knowledge, challenges, and future perspectives, this review offers insights into the molecular basis of nerve involvement in HNCs and the potential for novel therapeutic approaches.

## 1 Introduction

Carcinogenesis is a complex, multifactorial, and multistage process. As a result, it leads to the development and progression of malignancies. Furthermore, differently from purely genetic disease, cancer can be considered as an evolutionary and ecological process involving constant interactions between tumor cells and their environment (1–3). The increasing knowledge regarding the molecular processes which drive tumor growth and metastatic spread is paving the way for highly tailored and personalized treatment strategies which can, in turn, improve the long-term patient outcomes (1, 2, 4).

The current research on this topic is mainly focused on the concept of the tumor microenvironment (TME), which is considered as a local environment in which cancer cells and cancer stem cells (CSCs) develop (5). It consists of immune cells, blood vessels, cancer-associated fibroblasts, extracellular matrix (ECM), signaling molecules (6) and different types of nerves (7, 8). Nerve cells are active elements of the TME and the interactions between cancer cells and the host neuronal microenvironment through paracrine and electrochemical signaling have been recognized (7–13). In particular, an increase in the tumoral electrical activity has been described and explained as a consequence of the extensive functional connectivity between nerves and cancer cells, observed in various malignancies (14). As the peripheral nervous system is the source of many TME molecules, it may also play a significant role in cancer development and progression (5–9). Recent studies have identified a process of neoplastic expansion along nerves which is known as perineural invasion (PNI), meaning that some cancer cells disseminate to distant organs not only through lymphatic and blood vessels, but also invading the local nerves (7–9). PNI is a common finding especially in neurotropic malignancies, such as prostate and pancreatic cancer (7) and it has also been investigated in tumors of the head and neck (HN) region (15). As PNI is associated with worse clinical outcomes (9), scientists have put much effort into recognizing, defining, and also studying molecular and intercellular events behind the PNI process. However, the direct mechanisms underlying PNI are still not fully understood (7, 8, 15, 16). Besides PNI, several additional cancer-related neuronal phenomena have lately been recognized and the role of axonogenesis, neurogenesis, and neuronal transdifferentiation in carcinogenesis and tumor progression warrants further investigation (17–21).

HN cancers (HNCs) are a heterogenous group of malignancies occurring in the HN region. Worldwide, the incidence of HNCs in 2020 reaches approximately 900,000 cases, and 500,000 deaths annually (22). The main histological type of HNC is squamous cell carcinoma (SCC) (95% of HNCs cases), which develops from mucosal membranes of the upper gastrointestinal and respiratory tracts (23, 24). Several exogenic cancer risk factors such as tobacco use, alcohol consumption, human papillomavirus (HPV) infection, poor oral hygiene, and some occupations (such as roofer, cleaner, painter) have been identified for HNCs (23–26). Cancers affecting the HN region typically occur in elderly patients with heavy use of alcohol and tobacco, however, the prevalence in this group of people is decreasing (23, 27). On the other hand, the number of HPV-associated HNCs, especially among young people, is growing (22, 23, 27). HPV-positive HNCs tend to have a more favorable prognosis, with 5-year survival rates of 75%–80%, compared to less than 50% HPV-negative cases (23, 28). This disparity is partly due to increased sensitivity of HPV-positive tumors to chemotherapy and radiotherapy, as well as fewer distant metastases compared to HPV-negative cancers (29, 30). In contrast, HPV-negative tumors are associated with unfavorable genomic alterations, including *TP53* mutations and disruptions in cell-cycle regulators, contributing to their poorer outcomes (23, 31).

In addition, HNCs are among the most painful cancers, and they usually cause dysfunction and deformity in areas that are essential to patients’ daily functional and social activities. This means that HNCs are a determining factor in the decline in quality of life observed in these patients (23, 32).

The involvement of nerves in HNCs was first noted historically in 1862 by Neumann, describing PNI in a patient with primary SCC of the lower lip infiltrating the mental nerve (33). Subsequently, in the late 20th century, research further highlighted that HNCs could disseminate through the body via nerves at the microscopic level, and thus are not easily recognizable radiographically, meaning that exploring the nerve-cancer cell interactions in the carcinogenesis of HN tumors is essential (34, 35). These early findings highlighted that cancers could disseminate through the body via nerves at the microscopic level, and thus are not easily recognizable radiographically, meaning that exploring the nerve-cancer cell interactions in the carcinogenesis of HN tumors is essential. Compared to HPV+ tumors, HPV- HNCs are more densely innervated (21, 31), predominantly by sensory nerve fibers, and more painful (36, 37), which could be a consequent of the involvement of sympathetic, parasympathetic, and sensory innervation (38–42). PNI in HNCs is a complex phenomenon with significant clinical implications. In recent years, various histopathologic subclassifications of PNI in HNCs have been proposed (43–45). The interplay between nerves and HNCs, however, is intricate and not fully understood at molecular and structural levels. Further studies clarifying the role of cancer-nerve interactions in HNCs may facilitate the development of novel nerve-targeting treatment options or the repurposing of well-established drugs with potential anti-cancer effects (such as β-blockers) in oncology (46–54).

In this review, the aim is to report the current knowledge regarding the involvement of nerves in carcinogenesis, relevant molecular mechanisms focusing on HNCs. Furthermore, clinical significance of PNI, other nerve-related phenomena upon carcinogenesis and current therapies directions will be summarized.

## 2 Nerves participation in tumor microenvironment

TME is an extracellular matrix composed of CSCs, blood vessels, host cells (including endothelial cells, pericytes, immune cells, fibroblasts) and neurons, and macromolecules, such as collagen, glycoproteins, and elastin (6, 10). Constant interactions between malignant and nonmalignant cells result in TME development, which acts as the protumorigenic factor (5–7). In particular, nonmalignant cells provide signals for the uncontrolled proliferation of cancer cells (5, 55) and host cells supply a new vessel network and nutrition for cancer cells. Moreover, host inflammatory reactions enable the tumor to survive and play a role in the development of resistance to therapy (55, 56). In return, cancer cells influence TME host cells through paracrine signaling, matrix remodeling, and cell-cell interactions, resulting in a constant TME expansion and rebuilding which lead to tumor development, progression, and metastasis (8, 57).

**FIGURE 1:**
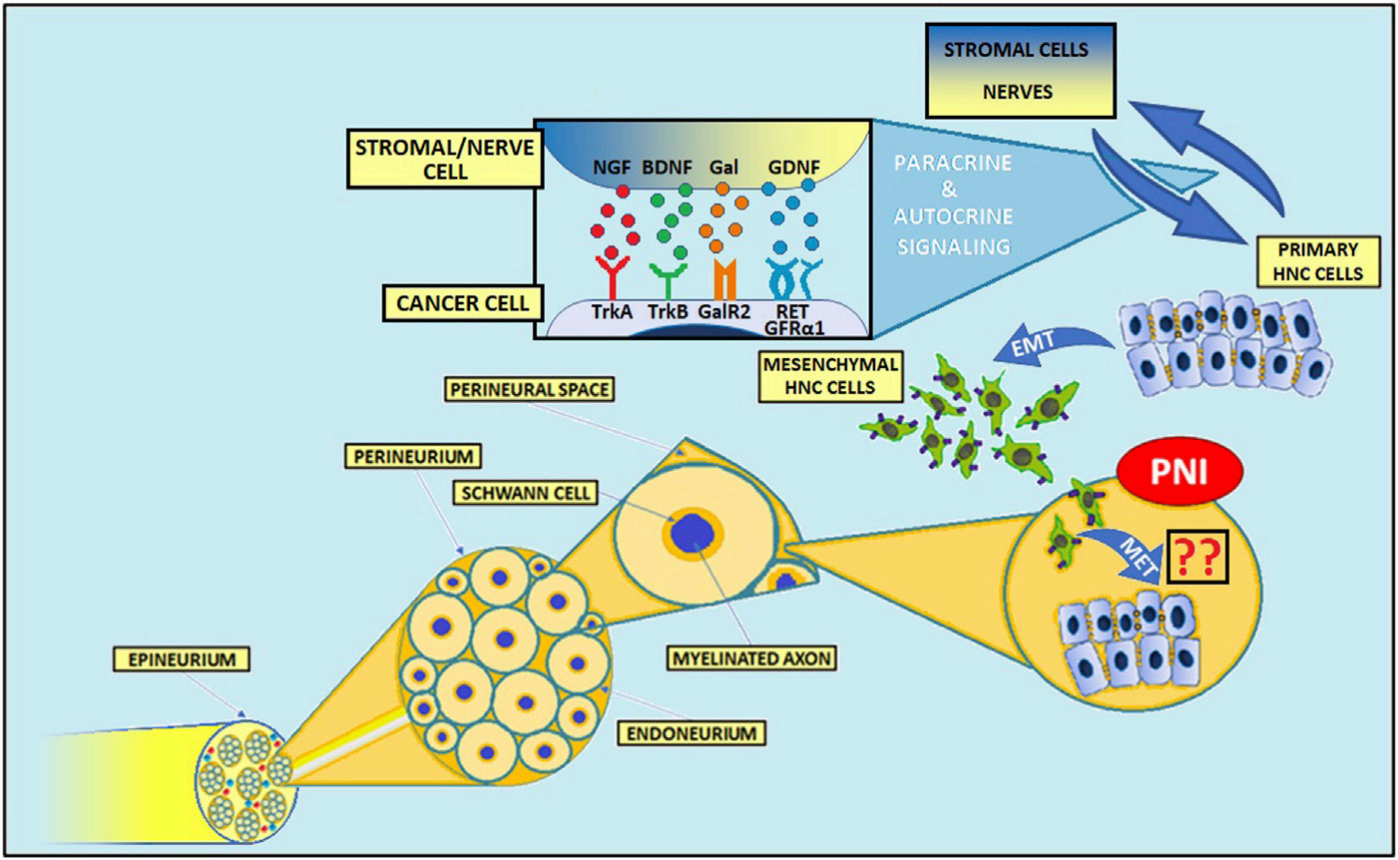
Structure of the peripheral nerve and mechanisms of PNI in head and neck cancers. The complex interactions between cancer cells, various stromal cells, and nerves contribute to the invasion of tumor cells into nerves. Numerous molecules, including NGF, BDNF, GDNF, and Gal play an important role in PNI pathogenesis. NGF, nerve growth factor; BDNF, brain-derived neurotrophic factor; Gal, galanin; GDNF, glial cell-derived neurotrophic factor; TrkA, tropomyosin receptor kinase A; TrkB, tropomyosin receptor kinase B; GalR2, galanin receptor 2; GFRα1, GDNF Family Receptor-α1; RET, rearranged during transfection; PNI, perineural invasion; EMT, epithelial-to-mesenchymal transition; MET, mesenchymal-to-epithelial transition.

As mentioned above, cancer’s niche consists of local nerves, which can constitute a favorable way to disseminate. Histologically, peripheral nerves consist of axon fascicles; groups of fascicles are surrounded by epineurium, which results from the fascicle transformation into connective tissue. In particular, endoneurium covers a single axon, lying upon layers of Schwann cells (SCs), which together work as a natural barrier for cancer dissemination. This means that infiltration through the nerves is challenging and neoplastic cells need to produce various mediators, such as neurotrophins, chemokines, cellular adhesion molecules, and some enzymes, such as metalloproteinases (MMPs), which favors extracellular matrix remodeling and collagen degradation (58, 59). Figure 1 shows the basic structure of the peripheral nerve.

The expression of MMPs is regulated by many factors, including interleukins, interferons, growth factors, TNF-α, and TGF-β (60). For instance, neural and cancer-derived nerve growth factor (NGF), binding to its receptor TrkA expressed on cancer cells, leads to p44/42 MAPK-mediated MMP-2 overexpression in pancreatic cancer (61–63). Also, glial cell-derived neurotrophic factor (GDNF)-RET signaling pathway activation stimulates both MMP-2 and MMP-9 expression in adenoid cystic carcinoma (ACC) of the salivary glands (64–67).

Another important element of TME is represented by SCs, which play a central role in nerve-cancer interactions (15). Their physiological functions include myelin formation in the peripheral nervous system and contribution to nerve repairing processes (57, 68, 69). During carcinogenesis, SCs can degrade the extracellular matrix (57). Also, in the presence of cancer cells, SCs upregulate the expression of glial fibrillary acidic protein (GFAP), typical of dedifferentiated SCs and involved in the nerve repair process (57). Notably, Deborde et al. revealed a close association between GFAP+ SCs and PNI occurrence in pancreatic adenocarcinoma, thyroid cancer, salivary duct carcinoma, and cutaneous SCC (57). Interestingly, the reduction of neural cell adhesion molecule 1 (NCAM1), a member of immunoglobulin superfamily cell adhesion molecules taking part in axon guidance and synapse formation, on SCs results in decreased cancer cell invasion (57, 70). Nevertheless, interconnections between NCAM1^low^ SCs and cancer cells are not fully eliminated, but cell recruitment and migration are significantly impaired (57). In a murine model, the increased interleukin 6 (IL-6) secretion from SCs, which results from the adenosine-mediated interplay between oral SCC and SCs, leads to hypertrophy and increased proliferation and migration of SCs (71, 72).

In summary, TME is involved in the main features of cancers - uncontrolled proliferation, migration, and invasion of cancer cells. The presence of cancer cells in the neighborhood leads to increased activity of host cells, which in turn could favor cancer cell growth. Moreover, SCs contribute to carcinogenesis, as they are an effective source of protumorigenic molecules for cancer cells. Due to the anatomy of the HN region, tumor cells have great access to the peripheral nerves. These nerves can be hijacked by tumor to modulate the TME. Finally, an in-depth understanding of complex interconnections between the various TME components is essential for the development of effective and personalized strategies to achieve improved cancer survival rates.

## 3 Perineural invasion in head and neck cancer

### 3.1 Definition, frequency, and subclassifications of PNI

#### 3.1.1 General information and definition of PNI

It is well-known that cancer cells can spread through the organism through lymphatic and blood vessels. Many recent studies emphasize, however, that the neoplastic invasion of peripheral nerves should also be acknowledged (9, 16, 73–78). PNI is a common pathological finding in various human cancers (9, 16, 73). Among cancers in the HN region, SCC and ACC are the most frequently nerve-invading malignancies (79). Moreover, HPV-associated HN SCCs are less likely to develop PNI (15).

Despite many years of research regarding PNI, its definition is still an open question. The first definition of PNI was proposed in 1985 by Batsakis et al., who considered PNI as the infiltration of tumor cells in, around, and through the nerves (9). Although there is no universal definition of PNI, the most commonly accepted one describes the PNI as a “tumor in close proximity to the nerve and involving at least 33% of its circumference or tumor cells within any of the three layers of the nerve sheath” (9).

#### 3.1.2 The prevalence and evaluation of PNI

The prevalence of PNI among cancers of the HN region reported in studies varies between 14% and 63% (80). PNI frequency differs depending on the tissue type, ranging from 27%–82% for cutaneous and mucosal types to 31%–96% for the ACC (81). Notably, PNI is more common in late-stage HN tumors (82). So far, histological evaluation is accepted as the best method for PNI recognition. To increase the detection rate of PNI in HNCs, the addition of immunohistochemical methods to the standard hematoxylin-eosin staining has been proposed (83–85). Also, utilizing deep learning and artificial intelligence (AI)-assisted approaches may provide a solution to the remarkable problem of intra- and inter-observer variability within the PNI identification in HNCs (86). While considering imaging techniques, the detection of cancer dissemination along nerves (perineural spread - PNS) is the most easily visible in MRI. MRI sensitivity for PNS detection in HNCs was divergent in different studies - in the latest, sensitivity reached 62% when using 1.5T MRI (87), whereas an earlier study that used 3T MRI, reached a sensitivity of 95% (88). Interestingly, radiomic features extracted from computed tomography offer a promising avenue for improving the detection, characterization, and prognostication of PNI in HN malignancies (87, 88). Radiomics is an emerging field that involves the extraction and analysis of a wide range of quantitative features from radiographic images, providing detailed insights into tumor characteristics, which could potentially aid in identifying and understanding PNI (89). Although symptomatic PNI occurs only in 30%–40% of patients, this percentage can be misleading as some early subtle symptoms can be overlooked (80). It should be noted that pretreatment pain may predict the presence of PNI in HN SCC patients (87).

In HN neoplasms, PNI primarily affects major nerves–CNV and CNVII, mainly due to the extensive spread of their fibers and abundant interconnections with other nerves (90, 91). Also, these nerves allow neoplasms to transit into intracranial space. Noteworthy, MRI is a gold standard to assess anatomical pathways of CNV and CNVII and review key landmarks for PNS detection in SCC, ACC, and other HNCs during the diagnostic process (90–92).

#### 3.1.3 The prognostic significance and subdivision of PNI

Several meta-analyses and systematic reviews have analyzed the prognostic role of PNI in various HN SCCs (93), ACCs (94), and parotid malignancies (95). Particularly, separate reports regarding PNI significance among HN SCC patients have been conducted in oral (96), oral tongue (97), and cutaneous malignancies (43). Accordingly, they concluded that PNI is associated with poor clinical outcomes across these cancers.

**FIGURE 2:**
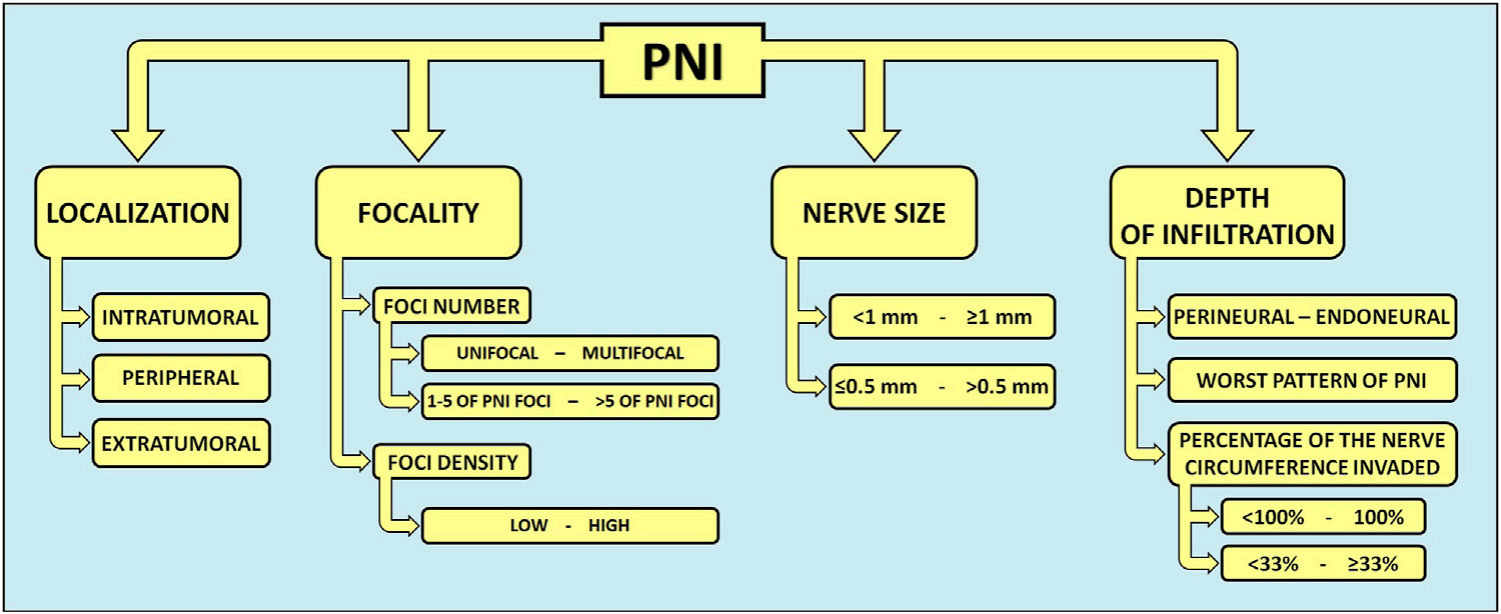
Quantitative and qualitative subclassifications of PNI in head and neck cancers. In HNCs, several graduations and descriptions of PNI have been proposed. A more precise description of PNI in the histopathological reporting of HNCs could guide the decision-making process for optimal cancer treatment. PNI, perineural invasion.

Nevertheless, numerous HNC-related reports indicate that, when defined using a simple dichotomous (present or absent) approach, the prognostic value of PNI is inaccurate and inconsistent (73, 98–101). Therefore, several quantitative and qualitative subclassifications of PNI have been proposed in recent years, taking into account: localization (intra-, peri-, or extratumoral) (44, 102–105), number of foci [unifocal/multifocal (45, 99, 105–107) and low PNI/high PNI groups with PNI foci 1-5 or >5, respectively (107)], foci density (low or high) (103–106), size of involved nerve [<1 mm or ≥1 mm (73, 99) and ≤0.5 mm or >0.5 mm (103)], gradation of PNI extent (percentage of the circumference of nerve involved by tumor: 100% or <100%) (103), depth of the tumor cells’ nerve invasion (44, 105, 106, 108), and worst pattern of PNI (WPNI): cancer cells encircling nerves <33%, ≥33%, or infiltrating into nerve sheaths (109) (Figure 2; Table 1).

**TABLE 1:** Summary of PNI subclassifications and their clinical relevance in various head and neck cancers.

PNI subclassification	Cancer type	Patient endpoints explored	Conclusion	Ref.
Localization (intra-, peri-, extratumoral)	Noncutaneous HN SCC, oral and oral tongue SCC	LRC, DSS, DFS, RFS, OS	Extratumoral PNI is associated with poorer prognosis, predicting worse DFS and/or OS compared to intratumoral PNI	(44, 102–105)
Number of Foci (unifocal/multifocal)	Oral and oral tongue SCC	Local failure, cervical lymph node metastasis, DSS, OS	Multifocal PNI predicts higher risk of local failure and disease-specific mortality. Moreover, multifocal PNI may be linked to a poorer prognosis irrespective of post-operative radiotherapy. It is also a stronger prognostic indicator than depth of invasion	(45, 99, 105–107)
Foci Density (low or high)	Oral and oral tongue SCC	DSS, risk of distant metastasis	High foci density is linked with a significantly increased risk of distant metastasis and a decrease in DSS	(103–106)
Size of Involved Nerve (<1 mm or ≥1 mm and ≤0.5 mm or >0.5 mm)	HN SCC, oral SCC	LF, DSS	Larger nerve involvement (≥1 mm) is associated with worse clinical outcomes, independent of other treatment factors	(73, 99, 103)
Gradation of PNI Extent (percentage of the circumference of nerve involved by tumor: 100% or <100%	Oral SCC	Survival outcomes, locoregional recurrence	Gradation of PNI extent was not predictive for survival outcomes on multivariable analysis	(103)
Worst Pattern of PNI (cancer cells encircling nerves <33%, ≥33%, or infiltrating into nerve sheaths)	Oral SCC	Lymph node metastasis, locoregional recurrence, distant metastasis, immune response	Higher worst pattern of PNI scores predict a more aggressive disease course, with higher rates of metastasis and recurrence, and poorer immune response	(109)
Endoneural (Intraneural) Invasion	Oral SCC, ACC, melanoma, cutaneous SCC	DSS, OS	ENI predicts poorer survival; PNI alone is not as predictive	(34, 35, 98, 100)

ACC, adenoid cystic carcinoma; DFS, disease-free survival; DSS, disease-specific survival; ENI, endoneural invasion; HN SCC, head and neck squamous cell carcinoma; LF, local failure; LRC, local-regional control; OS, overall survival; PNI, perineural invasion; RFS, recurrence-free survival.

Among HNCs, oral cancer has been the most extensively considered in studies focused on investigating the influence of histopathologic PNI sub-categories on patients’ outcomes. A recent systematic review concluded that PNI is generally a negative prognostic factor in oral cancer, in terms of locoregional recurrence, disease-free survival (DFS), and overall survival (OS) (96). However, identifying subgroups of oral cancer patients according to the severity of PNI could guide clinical decision-making and thus improve patients’ prognosis. For instance, multifocal PNI was a better predictor of the risk of local failure and disease-specific survival (DSS) than the depth of infiltration (106) - the acknowledged predictor for lymph node metastasis in oral SCC (110). Also, multifocal PNI elicits comparable effects to the presence of nodal metastases without extranodal extension on disease-specific mortality among oral SCC patients (106). Analogously, the quantification of PNI by focus number (no PNI, low PNI, and high PNI, with no, 1-5, and >5 PNI foci, respectively) was shown to predict cervical lymph node metastasis, poor 5-year DSS, and 5-year OS in early oral SCC (107). Moreover, Fu et al. introduced a new variable, WPNI, subdividing oral cancer patients into three types of WPNI (109). They presented that a higher WPNI score results in a more aggressive disease course, with more frequent lymph node metastasis, higher locoregional recurrence rate, and distant metastasis (109). Interestingly, patients with the highest WPNI showed the worst prognosis and impaired immune response, with a noticeable decrease in the total number of T cells, especially inhibitory CD3^+^CD8^+^ T cells, and B cells (109). In addition, Aivazian et al. study comprising 318 oral SCC patients, nearly one-third with PNI, provided insight into the plausible prognostic role of quantitative PNI characterization (99). Their findings indicate that patients with unifocal PNI display comparable outcomes to PNI-negative cases, regardless of the size of the involved nerve. At the same time, multifocal PNI was associated with worse clinical outcomes, irrespective of postoperative radiotherapy, especially when the involvement of nerves >1 mm in size was noted (99). Over a decade ago, Miller et al. categorized PNI according to its extent into intratumoral, peripheral, and extratumoral in HNCs (102). As their subcategorization was shown to be predictively relevant (102), more recent studies analyzed the association between PNI histological location and survival outcomes in oral SCC (44, 104). Two research teams have concluded that in comparison to intratumoral PNI, patients with extratumoral PNI carry a more unfavorable prognosis in oral SCC. On multivariable analysis, the presence of extratumoral PNI was associated with poorer 5-year locoregional control, DFS, and OS rates in Lee et al. research (44), whereas, in Park et al. study, extratumoral PNI was a significant predictor only for DFS among oral SCC patients (104).

Among all oral cancers, tongue SCC is the most prevalent malignancy (22, 111). Several authors have demonstrated that different PNI-related features can influence the prognostic value of PNI in patients with tongue SCC (97, 103, 105). Interestingly, Cracchiolo et al. reported that foci density, a novel histological characteristic of PNI, might have a prognostic utility in tongue cancer (103). Moreover, a subgroup analysis revealed that patients with high foci density PNI had a 19.4-fold increased risk of developing distant metastasis in comparison to an appreciable 6.4-fold increase in PNI-positive patients with oral tongue SCC (103).

It is worth noting that some authors distinguish an additional PNI-linked phenomenon - the endoneural (intraneural) invasion (ENI). It is defined as the presence of PNI with tumor cells invading into and/or with irregular destruction of the axon of the nerve bundles (98, 103). ENI has been acknowledged in various malignancies, including HNCs (34, 35, 112). It has been suggested that the distinction between PNI and ENI can affect the clinical approach to the patient, as confirmation of the fact that presence of the PNI or/and ENI can lead to disease recurrence and increased mortality (113). For instance, the occurrence of ENI histological examinations predicts poor prognosis in patients with ACC, whereas PNI occurrence does not significantly affect the OS (98). In accordance with these findings, a recent study on 235 patients with oral SCC revealed that the distinction between PNI and ENI could have a prognostic significance (100). Interestingly, PNI was identified in approximately 31.5 percent of all oral cancer patients, among which ENI concerned almost two-thirds of these cases. In a multivariate analysis, the occurrence of ENI was independently and significantly associated with poorer cancer-specific survival in patients with oral SCC. At the same time, the PNI presence was not correlated with any clinical parameters (100). In addition, the ENI occurrence has been linked with a higher frequency of either local or distant recurrence than in patients with PNI solely among pancreatic cancer patients who received the neoadjuvant therapy (112). Notably, DFS and OS were significantly shorter in patients with ENI, compared with PNI only (112). In contrast, an analysis of the influence of pretreatment facial weakness due to parotid gland cancers on PNI/ENI occurrence did not find any correlation between intra- or PNI and T and N tumor status. However, as one-third of resected facial nerves had no PNI/ENI evidence, the authors suggested that some patients could preserve the facial nerve if the decision regarding CNVII resection was based on intraoperative findings (114).

Recent evidence underscores the prognostic significance of PNI in HNCs, not only for its association with poor clinical outcomes but also for its link to cancer-associated pain. A novel study demonstrated that PNI independently predicts severe, activity-linked pain in patients with HN SCCs, highlighting its significant impact on daily functioning and quality of life. RNA sequencing analysis from The Cancer Genome Atlas (TCGA) revealed PNI-related disruptions in pathways associated with ECM, with several differentially expressed genes identified as potential molecular targets for cancer progression and pain management (115).

#### 3.1.4 PNI - conclusion and future directions

In summary, defining PNI remains challenging and requires clearer and more universal standardization. Addressing variability in histological PNI assessment is crucial for accurate diagnosis and prognostication. Advanced methods such as radiomics and AI-based technologies hold potential for reducing variability, improving detection rates, and expediting diagnosis in the context of PNI in HNCs (116). Simply defining PNI in a dichotomous manner (present or absent) is insufficient in the modern oncology era. Therefore, more precise evaluation and descriptions of PNI, with the indication of PNI stage, could be used to stratify patients with HNCs into different risk categories, requiring distinct treatment regimens. Also, there is a need for further studies comparing the clinical usefulness of PNI and ENI, as some authors question the role of PNI as the independent prognostic factor in HN malignancies (98, 100, 112). Finally, novel findings emphasize the need for comprehensive pain phenotyping and targeted interventions in PNI-positive HNC patients to improve both prognostic outcomes and pain control strategies (115).

### 3.2 Molecular mechanisms of PNI

There is growing evidence that interaction between peripheral nerves and cancer cells is essential for neoplasm development and dissemination (9, 16, 117). Cancers trigger PNI by influencing the secretion of various molecules, modulating numerous signaling pathways and altering the expression of different receptors and adhesion molecules (Figure 1). PNI is an invasive process, where genes associated with EMT, invasion, and metastasis are upregulated (118). It is crucial to observe that, in some terms, nerve-cancer crosstalk during PNI development is similar to nerves response to injury (9, 16, 117, 119). Cancer cells are able to damage nerves present in the TME through their demyelination and degradation, leading to the local inflammatory response and impairment of anti-tumoral immune activity (120). Consequently, nerve-cancer interactions may promote resistance to immune checkpoint inhibitors (120, 121). In some reports, the presence of PNI correlated with a positive epidermal growth factor receptor (EGFR) expression in HN SCCs (12, 121).

During PNI development, exosomes are contributing factors to the nerve-cancer crosstalk. They are a subtype of extracellular vesicles with a diameter usually ranging between 30 and 100 nm, which can contain DNA, RNA, lipids, metabolites, cytosolic and cell-surface proteins (73). Studies show that exosomes contribute to neoplastic transformation, tumor growth, epithelial-to-mesenchymal transition (EMT), metastasis, angiogenesis, and paraneoplastic syndromes (8, 21, 74). In addition, they presumably participate in tumor innervation, as their content released by exocytosis stimulates peritumoral neurites outgrowth (8). Noteworthy, exosome-induced innervation seems to be independent of neurotrophins (21). Madeo et al. revealed that exosomes derived from both HPV+ and HPV- HN SCCs may induce tumor innervation, a process additionally potentiated by ephrin B1-laden exosomes in their study (21). As an axonal guidance molecule, ephrin B1 can redirect nerve growth trajectory through its receptor. Moreover, salivary ACC-derived exosomes stimulate fibroblasts to produce NGF, eventually leading to PNI occurrence and cancer progression (77).

Recent studies have highlighted differences in innervation patterns between HPV+ and HPV- HNCs, showing that HPV- tumors are generally more densely innervated than their HPV+ counterparts (21, 31). The higher frequency of PNI in HPV-negative HN SCCs may be partly attributed to the prevalence of *TP53* mutations, which occur in over 85% of these tumors but are rare in HPV-positive cases (123, 124). Additionally, epigenetic modifications of genes contributing to cancer progression can lead to the PNI process in HNCs (125). In contrast, HPV-positive HN SCCs promote tumor innervation through distinct mechanisms, including *CCND1* gene (encoding cyclin D1) amplification influencing microRNA packaging in exosomes and the production of CD9^+^ exosomes containing oncogenes E6 and E7 (21, 126). Identifying biomarkers, such as *CCND1* amplification and exosomal oncogenes E6 and E7, is crucial for distinguishing subsets of HPV-positive patients, particularly those with densely innervated tumors, linked to aggressive behavior and potentially poorer clinical outcomes (17, 45). These insights could improve prognostication and guide the development of tailored treatments to modulate cancer-associated nerves and reduce tumor aggressiveness of certain HPV-positive HN SCCs.

Many studies reveal that the key element of PNI is the migration of axons to the tumor niche (9, 127–130). Among the diverse molecules present in the perineural niche, a few have a very well-researched role in PNI development. As they regulate neural growth and maturation, they are assumed to play a significant role in the perineural spread of neoplasms (9). The usage of these molecules can be seen figuratively as a tool for taking control of the local nervous system by cancer cells. Besides, tumor cells use upregulation of neurotrophins to become independent from the host’s paracrine system (7, 131–133). Having their own source of stimulating molecules, cancer cells are able to start the proteolytic enzyme-mediated infiltration of basement membranes and invasion of peripheral nerve fibers (9, 60, 61). The molecular mediators involved in PNI are discussed below.

#### 3.2.1 Nerve growth factor

NGF is a well-known neurotrophic factor and neuropeptide primarily involved in the development and survival of sympathetic and sensory neurons (134). The role of NGF in human malignancies has been recently emphasized (130, 132, 84).

Evidence from published studies indicates that within the TME of HNCs, NGF is predominantly secreted by cancer cells (135–138). This secretion occurs in both HPV-positive and HPV-negative HN SCCs, with NGF receptors not exhibiting HPV-specific distribution (139). For instance, a higher expression of NGF in the oral cancer tissue than in the surrounding tissues was observed (135). Also, NGF and its receptor, TrkA, are upregulated in oral SCC adjacent to areas of PNI (136). Similar findings were observed in ACC (138, 138). NGF binding to TrkA leads to the overproduction of MMP-2, an enzyme needed for tumor metastasis (90). In addition, oral tongue SCC research revealed that NGF expression in cancer cells correlates with PNI and lymph node metastasis (84). Furthermore, NGF upregulation correlates with larger tumor size, advanced clinical stage, greater tumor thickness, and close or positive section margin in oral SCC and ACC (84, 135, 140, 141). In oral and salivary cancers, NGF was shown to induce EMT, cell dispersion, and PNI, resulting in increased tumor aggressiveness via the activation of the PI3K/Akt signaling pathway (131). Importantly, NGF activating TrkA and NGFR facilitates PNI development and metastasis formation, and provokes pain in patients with oral SCC (142). Also, NGF modulates the expression of ATP receptors in mouse trigeminal ganglion cells, thereby enhancing pain sensation in the murine model of HN SCC (143). NGF may be also linked with cancer-induced cachexia (144).

Interestingly, salivary ACC is capable of producing exosomes promoting a fibroblast-mediated tumor neural invasion due to the expression of promigratory and proinflammatory molecules such as C-X-C motif chemokine ligand 12, neurotrophic receptor tyrosine kinases 1 and 2, neurotrophin 4, NGF, brain-derived neurotrophic factor (BDNF), and C-X-C chemokine receptor type 4 (77).

Zhang et al. study revealed constant interactions of cancer cells with SCs in pancreatic cancer (145). Pancreatic cancer cells can induce SC autophagy through NGF/ATG7 paracrine pathway. Subsequently, SCs reciprocally induce cancer cells’ autophagy and chemoattraction, resulting in SC proliferation, migration, and the promotion of PNI at the cancer site (145). Furthermore, Nodal, a protein playing a role in the activation of the differentiation process of embryonic tissues and neural development during embryogenesis (146), has been shown to influence the expression of NGF, BDNF, GDNF, and MMP-9, resulting in the enhancement of pancreatic cancer cell invasion ability, dorsal root ganglia (DRG) and synapse outgrowth, and hence the promotion of PNI (130).

The influential role of NGF signaling and its contribution to cancer-related features have led to an interest in anti-NGF treatment for its management (142, 144). Knowing that NGF blockade significantly reduced tumor proliferation, nociception, and weight loss in preclinical oral SCC models (143), anti-NGF is a promising treatment strategy to treat oral SCC progression, pain, and cachexia.

#### 3.2.2 Brain-derived neurotrophic factor

BDNF is believed to be overexpressed in various HNCs (142, 147). In health, it promotes the growth and differentiation of the nervous system (7). In head and neck malignancies, BDNF can be produced by both tumor and stromal cells, including cancer-associated fibroblasts (148, 149).

Ein et al. investigated the role of BDNF and TrkB in the *in vitro* model of oral tongue SCC. In their study, high levels of BDNF resulted in an increase in SCC and SC interaction and migration (150). Moreover, the use of high TrkB inhibitor (ANA-12) levels resulted in SCs dedifferentiation and migration (150). Interestingly, it also led to the formation of a border between Schwann and cancer cells, consequently slowing down the dissemination of cancer cells (150). In an *in vitro* co-culture oral SCC model, SCs and cancer cells carried crosstalk resulting in the migration of these cells towards each other (151). However, treatment with the TrkB inhibitor resulted in an SC-associated cancer cell dispersion (151). Therefore, SCs must be taken into account in understanding the PNI initiation. Another study exploring the cancer-SCs crosstalk demonstrated that BDNF/TrkB axis plays a crucial role in the PNI progression via the EMT induction in salivary ACC (152). Among intrahepatic cholangiocarcinoma patients, a high expression of BDNF correlates with the presence of PNI and lower survival rates (153). Interestingly, BDNF is likely to promote PNI in a dose-effect manner (153). Besides, BDNF/TrkB signaling may regulate tumor-induced facial hypersensitivity in oral cancer pain (154).

#### 3.2.3 Glial cell-derived neurotrophic factor

Another family of neurotrophins – GDNF – has been reported to possibly play a pivotal role in PNI pathogenesis. That family consists of GDNF, neurturin, artemin, and persephin. The binding of a GDNF member to its receptor - GDNF Family Receptor-α1 (GFRα1) - leads to the RET receptor activation (155). Alternatively, GDNF may also bind to the NCAM in a RET-independent manner (156). GDNF molecules promote survival, proliferation, migration, and invasion through effectors such as MAP kinase, AP-1 transcription factor, and MMPs in various cancer cell types (155, 157–160). GDNF is expressed in both HPV-positive and HPV-negative HN SCCs (161). Recent research suggested that GDNF has its role in PNI of HN SCC by promoting migration of these cells (160, 161). It was demonstrated that GDNF can induce the migration of HPV-positive SCC cancer cells (161). Additionally, sensory nerve-derived GDNF can promote HNC cells’ escape from NK cell control via JAK2-STAT1-mediated PD-L1 upregulation (160). Among patients with lacrimal ACC, GFRα1 and RET expression were positively correlated with PNI presence and cancer recurrence (162). Artemin, another member of the GDNF family, was found to be overexpressed in laryngeal SCC (163). Moreover, it corresponded with poor patient survival and advanced tumor stage (163).

In pancreatic cancer, Zhang et al. described the enhancement in CD74-dependent expression of GDNF. GNDF secretion resulted in a change in cell phenotype for more mobile, and subsequently, in PNI and outgrowth of DRG cells (164). Besides, soluble GFRα1 released from the DRG cells after binding with the cancer-derived GDNF activates the MAPK pathway, resulting in cells’ enhanced migratory potential and, in effect, nerve invasion by the cancer cells (64). Another pro-migratory pathway stimulated by GDNF in human salivary ductal carcinoma involves the GDNF-RET-β-Pix-Cdc42 signaling cascade (165).

#### 3.2.4 Galanin

Galanin (Gal), a peptide derived from both sensory neurons in the dorsal root ganglia and cancer cells, contributes to the HN SCC development (128, 166, 167). Generally, Gal receptor 1 (GalR1) is involved in tumor suppression and exerts antiproliferative actions, whereas activation of GalR2 induces antiproliferative or proliferative effects in HN SCCs (168). Epigenetic mechanisms regarding Gal and its receptors are related to HNC tumorigenesis (166, 168). Likewise, Gal and GalR1/2 promoter methylation status may serve as a potential biomarker for predicting clinical outcomes in patients with HN SCCs and salivary duct carcinoma, a rare and aggressive parotid gland malignancy (166, 168). It was also demonstrated that Gal overexpression correlates with poorer OS in HN SCC (170). Accordingly, high Gal/GalR2 levels were associated with a decreased OS of salivary ACC patients (171).

Activation of the Gal/GalR2 signaling results in cell proliferation, survival, angiogenesis, and immunosuppression in HN SCCs (170, 172, 173). Conversely, GalR2 may inhibit tumor cell proliferation and induce caspase-3-dependent apoptotic mechanisms (174). Interestingly, GalR2 activation leads to the increased secretion of its ligand, Gal, in a positive autocrine feedback loop in this malignancy (128). In response, cancer-derived Gal promotes neuronal outgrowth into the TME and invasion of cancer cells into nerves (128). Particularly, nerve-derived Gal could stimulate the PNI through the EMT process in this cancer (171). Therefore, targeting the GalR2-induced pathway or blocking Gal offers a promising clinical strategy for disrupting the neural-tumor crosstalk in PNI, potentially improving treatment outcomes of HNC patients.

## 4 Axonogenesis, neurogenesis, and nerve transdifferentiation

PNI and axogenesis are two different processes: the first refers to the dissemination of cancer cells by nerve fibers, while the latter is described as a nerve outgrowth into the tumor niche (21). They both contribute to cancer growth, development, and metastasis (7, 175). Another nerve-related phenomenon is neurogenesis - an outgrowth of neuronal cell progenitors in the cancer niche, derived from CSCs or cells migrating from the central nervous system (18–20, 176). As an outcome, axonogenesis and neurogenesis may lead to nerve outgrowth at the tumor site, measured by nerve density, usually defined as the number/area of nerves divided by the total area analyzed (177). Although not fully understood, the PI3K-mTOR pathway, affected by the *TP53* mutation status, may play a role in regulating nerve density within the TME of HN SCCs (178). In most reports, including HNCs, higher nerve density correlated with more advanced cancers with a worse clinical prognosis (17, 42, 179). Noteworthy, the interactions between stromal cells, epithelial cells, and nerves constitute the natural base of this process (8, 21). Under physiological conditions, this interplay is coupled with processes followed by nerve injury (58). Many recent studies, especially those investigating prostate cancer, show that axonogenesis precedes and facilitates PNI (175). In addition, the occurrence of neural progenitors within the TME initiating cancer-related neurogenesis during cancer development has been detected in prostate cancer and hepatocellular carcinoma (18, 180). Notably, in hepatocellular carcinoma, neural progenitors predominantly express parasympathetic features (180).

Data on neurogenesis in HNCs are currently limited. While the study by Amit et al. primarily investigated the effects of p53 loss on neuronal reprogramming, it did not explicitly demonstrate neurogenesis as defined by the migration of cancer stem cells from the central nervous system. Amit et al. reported that loss of p53 in HNC led to increased axonogenesis and recruitment of adrenergic fibers, which were associated with enhanced cancer cell proliferation in HPV-negative oral SCC (17). Additionally, in a mouse model of HPV-negative oral SCC, cancer cells were shown to transfer microRNA molecules to adjacent neuronal cells, resulting in a sensory-to-adrenergic switch in peripheral nerves and promoting tumor progression (17). Although this study does not directly address neurogenesis from central nervous system-derived progenitors, it provides valuable insights into the mechanisms by which tumor cells can influence and reprogram the neuronal environment to support cancer progression.

Of note, CSCs, a population of cells found in many common cancers, including HNCs (20, 181), can switch into tyrosine hydroxylase-positive cells, characteristic of sympathetic neurons (182). Moreover, CSCs may convert to ACh-producing cells - a pattern suggestive of parasympathetic neurons (182).

Tumors secrete various neurotrophins to promote axonogenesis, such as NGF, BDNF, neurotrophin-3 (NT3), and NT4/5 that activate the process via the tyrosine kinase receptors expressed in nerve terminals (16). Another group of molecules proved to play a role in axonogenesis are semaphorins, the family of chemorepulsive axon guidance molecules, and their primary receptors, neuropilins and plexins (76, 183). Interestingly, Semaphorin 4F-NF-kB interactions, resulting in neurogenesis in prostate cancer, facilitate metastasis formation (184). In ACC, EphA2/ephrin A1 expression has been found to be correlated with PNI and vascular invasion (38). Also, their overexpression was associated with an increased potential for metastasis development and poor clinical outcome (38).

The next molecules involved in axonogenesis are cadherins and immunoglobulin-like cell-adhesion molecules (Ig-CAM) (7, 185). It has been shown that NCAM can accelerate PNI and axonogenesis (7). Also, the Overexpression of NCAM1 can be observed together with E-cadherin loss during the EMT (57). Controversially, studies on SCCs of the HN show that NCAM does not take part in PNI processes (186).

## 5 Innervation modifications in head and neck cancers

The autonomic nervous system provides innervation to almost every organ in the human body. It consists of two functionally complementary parts - sympathetic and parasympathetic, altogether playing an active role in preserving homeostasis (187). The most characteristic feature of the innervation in the HN region is the twelve pairs of cranial nerves, which provide a very rich sensory innervation (117). Given the rich innervation of the HN region, cancers arising in this area have substantial opportunities to exploit the nervous system for enhanced development.

The HN region exhibits significant site-specific variations in nerve types, which can markedly influence the biology of cancers in these areas. For instance, sensory nerves, primarily via the trigeminal nerve, are more prominent in the oral cavity and tongue, while parasympathetic nerves dominate in the parotid gland, and a mixed innervation is found in the larynx and nasopharynx (189–190). Understanding these differences is essential for elucidating how nerve-cancer interactions vary across different HNCs, potentially guiding more targeted therapeutic approaches.

Importantly, the involvement of distinct parts of the autonomic nervous system can lead to various effects during carcinogenesis. The influence of sympathetic, parasympathetic, and sensory nerves on HNCs will be discussed in detail below.

The distinct involvement of various components of the autonomic nervous system can lead to diverse effects during carcinogenesis. The roles of sympathetic, parasympathetic, and sensory nerves in the progression of HNCs will be discussed in detail below.

### 5.1 Sympathetic innervation

#### 5.1.1 Background

Sympathetic innervation arises from the cervical sympathetic chain, formed by the neurons from spinal cord segments (from T1 to L2). In the cervical part - superior, middle, and inferior - cervical ganglia are formed (191, 192). In general, preganglionic neurons synapse and activate nicotinic receptors on postganglionic neurons utilizing primarily acetylcholine (ACh), while postganglionic fibers predominantly use norepinephrine (NE) to influence the target organs. Both adrenaline and NE bind to and activate adrenergic receptors, further subdivided into α1-, α2-, β1-, and β2-adrenoreceptors. In addition to the main quick-acting transmitter, cotransmitters with a prolonged effect like ATP and neuropeptide Y (NPY), are secreted (193, 194).

Sympathetic nerves are implicated in promoting tumorigenesis across various malignancies, including prostate cancer, pancreatic cancer, and HNCs (16, 196–197). Adrenergic nerves release NE, which interacts with different adrenergic receptors to drive and modulate multiple processes related to carcinogenesis (16, 59, 195–199). For instance, NE-mediated upregulation of the β2-adrenoreceptor in endothelial cells induces angiogenesis in prostate cancer (16, 195). In an orthotopic murine breast cancer model, NE promoted migration and metastasis through β2-adrenergic activation of CREB and NF-kB family transcription factors (196, 197). Similarly, in pancreatic cancer, NE derived from sympathetic nerves causes overexpression of NGF and MMPs, leading to PNI development (200).

#### 5.1.2 Clinical observations and contrasting effects of sympathetic nerves in HNCs

Patients with HNCs exhibit higher plasma NE and adrenaline levels than patients without cancer (199). Importantly, stress hormones (NE and cortisol) were shown to promote DNA damage in oral keratinocytes, thus predisposing them to cell malignant transformation (201). In salivary ACC, a tissue concentration of NE and density of β2 receptor on cancer cells surface correlated with higher cells’ migration by stimulating the upregulation of N-cadherin and metalloproteinases, and downregulation of E-cadherin (39). Moreover, β2-receptor overexpression is associated with a higher TNM stage and PNI in this malignancy (39). In oral SCC, the overexpression of β2 receptors has been correlated with a higher clinical stage of the disease and with preoperative lymphatic metastasis (202). Furthermore, in a breast cancer model, sympathetic innervation was also shown to take part in tumor outgrowth and suppression of adaptive immune cells (198).

In contrast to the abundant cancer-promoting findings, Bravo-Calderón et al. demonstrated that NE can enhance the anti-proliferative and anti-invasive properties of cancer cells via the β2-adrenoreceptor activation in the oral SCC model (203). Consistently, novel findings revealed a cancer-protective function of sympathetic nerves during the development and progression in the murine model of pancreatic cancer mediated by the local suppression of cancer-promoting and immunosuppressive CD163+ macrophages in the TME (204).

#### 5.1.3 α-Adrenergic receptors and HNCs

When it comes to the α-adrenergic receptors, various reports indicate that α-adrenergic stimulation exerts pro-survival effects in several cancers (46–49). As for the role of α-adrenoreceptors in HNCs, data are scarce. Nevertheless, a single report revealed that the α1-adrenergic receptor can serve as a potential diagnostic biomarker in oral SCC (50). In their study, Gholizadeh et al. reported a higher concentration of α1-adrenoreceptors in saliva samples of patients with cancer of the tongue, buccal mucosa, and gingiva in comparison to healthy controls (50).

#### 5.1.4 Impact of sympathectomy and β-blockers on HNCs

While it is becoming increasingly evident that β receptors exhibit a versatile role in cancer biology, it is worth exploring whether β-blockers may be beneficial in cancer treatment. For instance, in oral, laryngeal, nasal, and pharyngeal cancer cell lines, propranolol, a non-selective β-adrenergic blocker, significantly decreased the viability and proliferation of cancer cells (210). In rat model, propranolol consumption reduced the occurrence of oral SCC, its invasiveness, and the release of pro-inflammatory cytokines (40). It also inhibited cell proliferation, migration, and metastasis in HPV-associated HN SCC (41), and selective β2-blockers improved survival in the orthotopic oral SCC murine model (202). Contrarily, prior administration of propranolol abolishes the anti-tumorigenic effects of NE in oral SCC cell lines (203). Similarly, propranolol administration and chemical sympathectomy attenuated the antitumor activity mediated by the enriched environment in the rodent pancreatic cancer model (42). In clinical studies concerning HNC patients, β-blocker use was associated with worse OS, DSS, and DFS (211–214). Finally, β-blockers appear to have no preventive effect on HNCs (214).

These observations raise the question about the possible utilization of sympathectomy as a potential treatment or at least a method to suppress cancer development. To date, conflicting findings regarding the effects of sympathectomy have been reported (51, 52, 204, 205). For instance, sympathectomy inhibits tumor growth, invasiveness, and density of lymph vessels in tongue carcinoma in rats (51, 52). Contrarily, a recent study revealed accelerated tumor growth, increased metastatic spread, and poorer disease outcomes in sympathectomized mice with pancreatic cancer (204). Moreover, the outcome of the sympathetic denervation is likely time-dependent (204, 205). The heterogeneous effects of sympathetic denervation could be explained by the existence of distinct subtypes of sympathetic nerve fibers within the TME, taking part in different signaling pathways, either cancer-promoting or tumor-suppressing (206–209). As the presence of molecularly distinct sympathetic nerve fibers in a cancer-specific context has not been well-characterized, it is of great significance to investigate this matter.

#### 5.1.5 Conclusion

**FIGURE 3:**
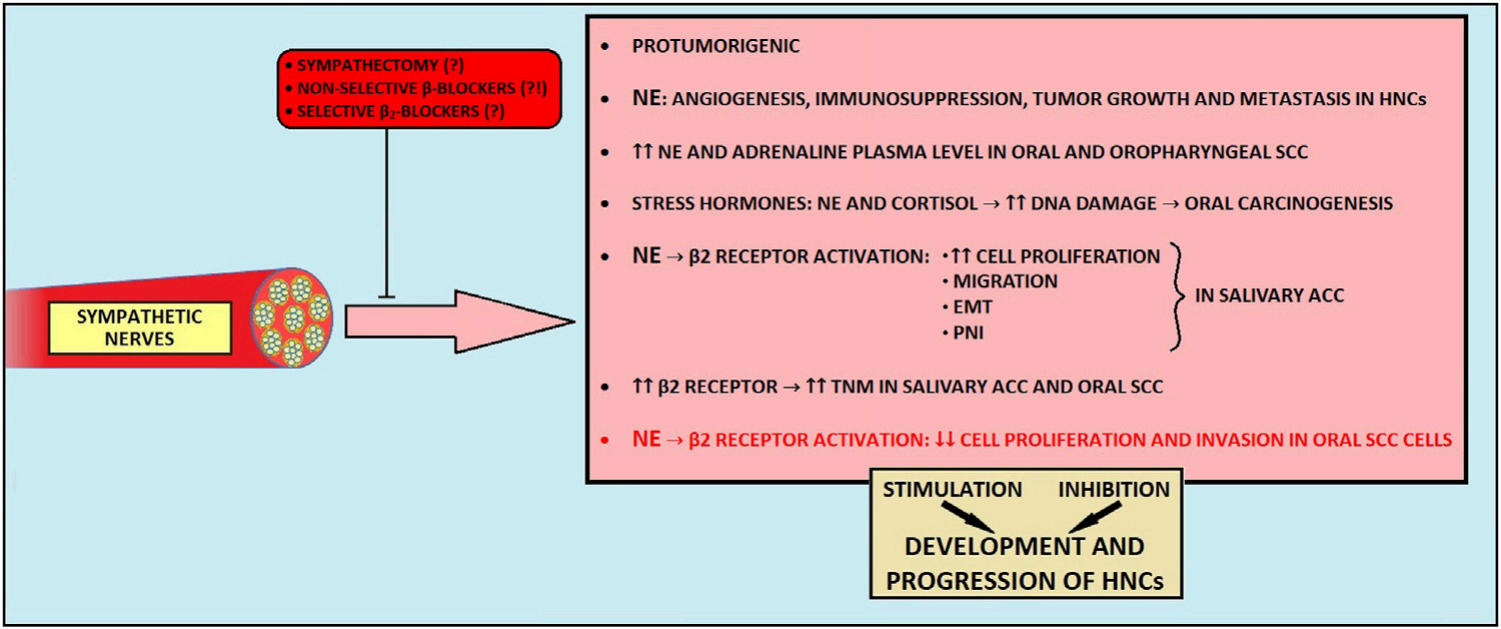
The influence of sympathetic nerves on development and progression of head and neck cancers. EMT, epithelial-to-mesenchymal transition; HNCs, head and neck cancers; NE, norepinephrine; PNI, perineural invasion; SCC, squamous cell carcinoma.

In summary, the crosstalk between sympathetic output, HNCs, and its molecular mechanisms are not fully elucidated (Figure 3). It is possible that sympathetic nerves, at the same time, play a Janus-like role by limiting and promoting the development and progression of HNCs. Given that the adjuvant use of non-selective beta-blockers in cancer therapy has yielded ambiguous results and that it is also burdened with cardiovascular side effects, their use as part of the combination therapy on a wider scale could be clinically unacceptable (215). Future research should focus on identifying distinct sympathetic nerve subtypes within the tumor microenvironment and their specific effects on cancer progression. Additionally, exploring the timing and selective targeting of β-blockers, along with alternative therapeutic strategies, may offer more effective and safer approaches.

### 5.2 Parasympathetic innervation

#### 5.2.1 Background

The HN region receives parasympathetic innervation from four nuclei situated within the brainstem. After exiting the brain, the parasympathetic fibers from each nuclei synapse in the peripheral ganglia near the target organ. Postganglionic neurons use ACh as the effector neurotransmitter acting on muscarinic (M1-M5) receptors and nicotinic receptors, consisting of numerous homomeric and heteromeric pentameric structures, made by α and β subunits (209, 216). Regarding cotransmitters, vasoactive intestinal polypeptide (VIP) and ATP are concurrently released from parasympathetic nerve fibers along with ACh (193, 194). Cholinergic transmission is regulated by cholinesterases effectively hydrolysing ACh, hence terminating its activity (217). There are two types of cholinesterases in humans - acetylcholinesterase (AChE), existing in three isoforms, and butyrylcholinesterase (BChE) (218, 219). They are present in various tissues, including synapses.

Numerous studies have reported the role of neuronal-derived acetylcholine in cancer growth, progression, and development of distant metastasis (220–222). In general, ACh produced by the parasympathetic nerves is able to enhance cell proliferation, migration, EMT, angiogenesis, and other malignant characteristics (182, 198). A growing body of research indicates that different receptors are involved in these processes (198, 222–224). Also, cancer cells are believed to have evolved diverse strategies to maintain appropriate ACh concentrations within the TME (225). The current overview and future perspectives of cholinergic signaling in HNCs will be presented in this section.

#### 5.2.2 Role of nicotinic receptors in HNCs

Various studies investigated the role of nicotinic receptors in HNCs. For instance, tobacco-derived nitrosamines and nicotine activate nicotinic acetylcholine receptors (nAChRs), facilitating carcinogenic processes (216, 226). Shimizu et al. recently demonstrated that nicotine, through activation of α7 nAChR, one of the most crucial cancer-stimulating nicotinic receptors (227), stimulates phosphorylated-EGFR accumulation and protein kinase B activation, therefore causing increased cell proliferation and migration in HN SCC cells (228). Moreover, nicotine-mediated stimulation of this receptor resulted in lymph node metastasis formation in the murine model (228). Also, α7 nAChR activation by nicotine promotes the oral SCC cells’ proliferation and migration through EGFR-mediated MEK/ERK and phosphatidylinositol-3 kinase/AKT pathways (229). Nicotine stimulation of the α7 subunit can also activate the JAK2/STAT3 axis (230). Interestingly, metformin and dextromethorphan can inhibit α7 nAChR/JAK2/STAT3/SOX2-mediated esophageal SCC progression (231). It should be noted that widespread STAT3 overexpression correlates with the aggressiveness of HNCs (53, 232–234). Besides, as STAT3 signaling contributes to chemoradiotherapy resistance and immune escape in numerous epithelial cancers (235, 236), it may constitute a promising target for novel resistance overcoming and immunity-boosting cancer treatments. A growing body of evidence links nicotinic stimulation of α7 nAChR with resistance to various chemotherapeutic agents. For instance, nicotine-mediated α7 nAChR stimulation may contribute to cetuximab, (an anti-EGFR monoclonal antibody) resistance in HN SCC (228) and cisplatin-resistance in oral SCC (237). Also, α7 nAChR mediates resistance to sorafenib in hepatocellular carcinoma (238) and paclitaxel in triple-negative breast cancer (239). In addition to these findings, α5 nAChR-mediated E2F signaling pathway activation may lead to radioresistance among patients with oral SCC (240). Therefore, novel subunit-specific inhibitors targeting nAChRs (including α7 nAChR) could help achieve an increased sensitivity to therapeutic regimens in various HNCs.

In The Cancer Genome Atlas (TCGA) cohort data, among plenty of upregulated nAChRs, highly expressed α5, α9, and β4 subunits were associated with poor prognosis in smoking patients with HN SCCs (224). Moreover, a novel prognostic signature based on the expression of these three subunits proved to be an independent prognostic factor for OS (224). Surprisingly, a small study suggested that α3, α5, and α7 subunits might have no prognostic value in HNCs (223). Interestingly, one study reported a possible uneven distribution of nAChRs within malignant tissues of the HN region (241). Moreover, overexpression of α1 and downregulation of α3 and α7 at the mRNA level were detected in laryngeal SCC (241). Interestingly, Chuang et al. recently suggested that inhibition of the β4 nAChR with varenicline, an FDA-approved medication used for smoking cessation and a potential β4 subunit inhibitor, could reduce metastasis in smoking HN SCC patients with upregulated β4 nAChR (54). In a Brazilian population study, the polymorphism in the gene encoding α5 nAChR subunit was associated with the intensity of cigarette consumption, indirectly influencing the HNC risk (242).

#### 5.2.3 Muscarinic receptors and HNCs

In contrast to nicotinic AChRs, the role of muscarinic receptors in HNC tumorigenesis remains understudied. In some malignancies, muscarinic receptors have been described as the proliferation activating factor (222, 243, 244). The most extensively studied mAChR with regard to cancer research is the muscarinic M3 receptor (244–246). M3 receptor plays a crucial role in tumorigenesis in various cancers, including gastric, colorectal, and lung malignancies (247, 248). Hence, the utilization of M3 mAChR as a potential drug target has been proposed (249–251). The expression of the M3 receptor was elevated in HN SCC and ACC cell lines, with higher M3 mAChR expression in SCC cells than in ACC cells (249). In contrast, salivary gland adenocarcinoma and submandibular cancer cells exhibited decreased M3 mAChR expression levels via hypermethylation of CpG islands located in the promoter region of the gene encoding M3 mAChR protein (252). Interestingly, Sun et al. revealed that acacetin, a plant-derived flavonoid compound, may induce cell apoptosis of HN SCC cells *in vitro* by cytochrome c-mediated caspase 3 activation and M3 mAChR-related calcium signaling (249). Also, high expression levels of M5 and M1 mAChRs have been described in submandibular cancer cells (253). Their activation leads to the proliferation of submandibular cancer cells via stimulation of phospholipase C/nitric oxide synthase/arginase and phospholipase A2/cyclooxygenase pathways (253). In addition to these findings, the M4 mAChR activation may induce migration of oral SCC cells via stimulation of the Src family kinases/extracellular signal-regulated kinase 1/2 axis (254).

#### 5.2.4 Cholinesterases and ACh levels in HNCs

Since ACh promotes cancer, it is reasonable to assume that its levels should be maintained in the TME through increased synthesis and reduced activity of ACh-degrading enzymes (219). Consequently, increased ACh levels can cause excessive activation of ACh receptors (221). Reduced AChE activity has been observed in laryngeal SCCs, while both AChE and BChE activity were decreased in HN SCCs (13, 221) with tobacco and alcohol use linked to diminished activity of AChE in laryngeal SCC (13). Moreover, smokers with HN SCCs showed diminished mRNA expression of ACh-degrading enzymes (221). Lower AChE activity in HN and laryngeal SCCs correlated with poorer prognosis, shorter OS, and may act as an independent prognostic marker, suggesting that lifestyle factors may decrease ACh-hydrolyzing enzymes’ levels, worsening outcomes (13, 221). In oral SCC, findings on ACh-degrading enzymes have been mixed (254–258). Most studies report decreased BChE serum levels (254–256), whereas one found elevated BChE levels in blood (257). In addition, BChE activity appears to decline with the advancement of tumor stage in oral SCC (254, 255). Noteworthy, BChE serum levels could also serve as a potential prognostic biomarker in HNC patients undergoing radiotherapy (256).

#### 5.2.5 Non-neuronal sources of Ach

Unlike traditional assumptions that cholinergic signaling requires parasympathetic innervation, novel findings indicate that ACh can be secreted from non-neuronal tissues, including immune cells, within the oral SCC microenvironment (257). In an orthotopic oral SCC models investigating the role of ACh-secreting CD8^+^ T cells, impaired ACh-muscarinic signaling led to accelerated tumor growth and reduced tumor-infiltrating lymphocytes (TILs) in the TME (257). Moreover, it has been suggested that Ach-muscarinic axis is crucial in promoting CD8^+^ T cell infiltration and maintaining memory T cells in the TME, thereby facilitating sustained anti-tumor immune responses in oral SCC (257).

#### 5.2.6 Conclusion

**FIGURE 4:**
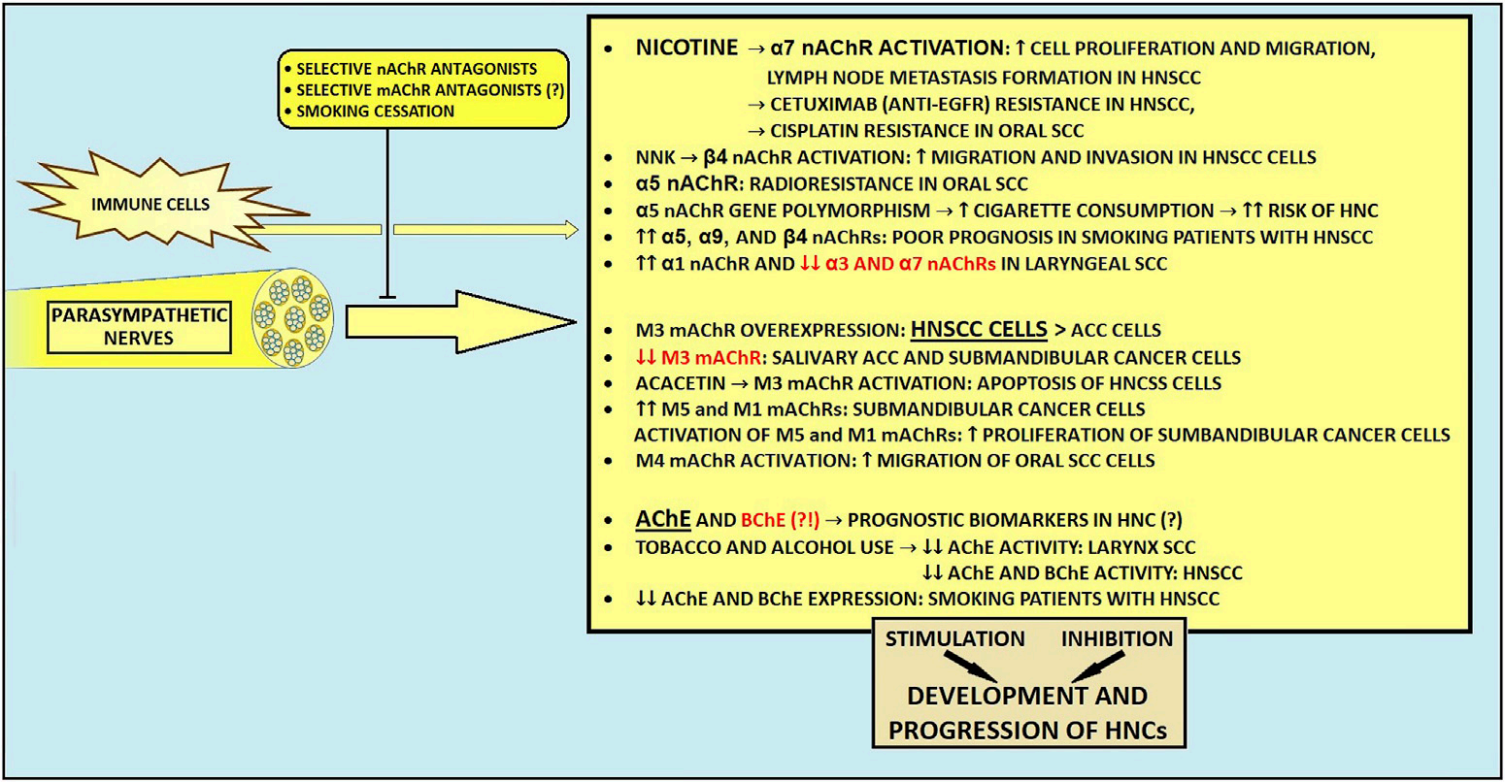
The influence of parasympathetic nerves on development and progression of head and neck cancers. ACC, adenoid cystic carcinoma; AChE, acetylcholinesterase; BChE, butyrylcholinesterase; EGFR, epidermal growth factor receptor; HNCs, head and neck cancers; HNSCC, head and neck squamous cell carcinoma; mAChR, muscarinic acetylcholine receptor; nAChR, nicotinic acetylcholine receptor; NNK, Nicotine-derived nitrosamine ketone.

Taken together, ACh-activated muscarinic and nicotinic AChRs are related to carcinogenesis, and some particular types of them could become potential markers of clinical prognosis (Figure 4). Investigating how ACh affects immune cells within the TME could reveal new strategies to enhance anti-tumor immunity and overcome immune evasion. Given ACh’s role in promoting cancer cell growth, further research into ACh-degrading enzymes and their impact on carcinogenesis is warranted. Developing selective inhibitors for cholinergic receptors and evaluating their clinical effectiveness could provide new therapeutic options. Additionally, the discovery of non-neuronal sources of ACh suggests the need for research into how these sources influence tumor progression and immune responses. Identifying biomarkers related to cholinergic signaling for predicting disease outcomes and treatment responses could also enhance personalized treatment strategies.

### 5.3 Sensory innervation

#### 5.3.1 Background

The HN region is richly innervated by sensory nerve fibers. The two main cotransmitters taking part in the modulation of sensation transmission are calcitonin gene-related peptide (CGRP) and substance P (SP) (194). SP contributes to neuroinflammation (261), whereas CGRP has a well-documented anti-inflammatory effect (262). Moreover, capsaicin activates type C and type A small capsaicin-sensitive sensory fibers via the transient receptor potential vanilloid 1 (TRPV1) and ankyrin 1 (TRPA1) receptors (263, 264). These fibers contribute to pain sensation and neuroinflammation by releasing SP and CGRP (265, 266). Also, these nerves take part in immune responses and promote tissue repair (267, 268).

Apart from the recognized role of parasympathetic and sympathetic nerve fibers within the TME, some studies have also reported the participation of the sensory fibers in carcinogenesis (36, 269). Nevertheless, the role of sensory nerves is unclear, and, it is thought that depending on the stage of development or the tumor aggressiveness, sensory input can either enhance or inhibit the tumor outgrowth (117). To date, CGRP and SP seem to be the most prominently reported sensory-related molecules influencing the cancer microenvironment.

During cancer development and PNI, sensory fibers become damaged and degenerated through disorganization and loss of myelin and axons (262, 270). Concurrently, cancer stimulates the generation of new morphologically altered neurons, which can contribute to abnormal nerve activity and increased pain sensitivity (271–273). In recent studies it has been shown that patients with peripheral nervous system infiltration experience increased pain (23, 273, 274). Interestingly, studies on gastric and pancreatic malignancies and bone metastasis described the inhibition of carcinogenesis and lesser pain sensation after the destruction of sensory fibers (255, 274).

As previously noted, HPV- tumors are more densely innervated than HPV + HN SCCs (21, 31). Intriguingly, patients with HPV-negative HNCs have a higher prevalence of pain compared to patients with HPV-related tumors (37, 275). Importantly, TRPV1-expressing nociceptive sensory fibers constitute the majority of intratumoral nerves in both HPV+ and HPV- HN SCCs (117). Also, α-CGRP-positive sensory nerve fibers may comprise approximately 10% of all nerves found in the TME of HN SCCs (276). Madeo et al. demonstrated a probable mechanism contributing to tumor innervation, where cancer-derived exosomes can induce an outgrowth of TRPV-positive sensory nerves in HN SCCs (21). Noteworthy, intratumoral fibers present within the TME, with a significant share of TRPV1-expressing nerves, establish potential functional connectivity, leading to increased electrical activity in the tumor bed (271). In a murine oral SCC model, sensory nerves differentiated into adrenergic neo-neurons through loss of *TP53*, leading to tumor progression (17). Parallelly, sensory neural input can contribute to the immunosuppressive microenvironment in HNCs through the release of CGRP (277). At the same time, surgical ablation of sensory nerves was demonstrated to abrogate tumor growth (17) but also to improve the efficacy of anti-PD-1 immunotherapy through downregulated TGFβ signaling and decreased PD-L1 expression in HN SCCs (278).

#### 5.3.2 Calcitonin gene-related peptide

CGRP is the predominant neurotransmitter in trigeminal ganglia neurons innervating the tongue (278). It exists in two isoforms: α-CGRP, produced from the *CALCA* gene, is the primary form present in both the central and peripheral nervous systems, while β-CGRP, derived from the *CALCB* gene, is predominantly found in the enteric nervous system. CGRP secreted from nerve endings exerts paracrine effects, leading to the enhancement of vasodilation-mediated tumor angiogenesis in oral SCC (280). Some studies reported that CGRP could stimulate cancer development through metabolic reprogramming (14, 281). Importantly, α-CGRP produced by melanoma-innervating nerves derived from cancer-induced axonogenesis may induce an immunosuppressive TME, which leads to impaired function of CD4^+^ and CD8^+^ T cells (14, 272, 273). Moreover, melanoma-infiltrating TRPV1^+^ nociceptors overexpressing *CALCA* and *Trka* - a high-affinity receptor for NGF - may promote cancer-induced pain hypersensivity (272, 275). Recent findings also reveal that nociceptive TRPV1-positive nerves infiltrating an adenosine-rich oral SCC microenvironment release α-CGRP upon stimulation of adenosine A_2A_ receptor on trigeminal ganglia, thereby contributing to tumor progression (282). Noteworthy, using istradefylline, a clinically available A_2A_ receptor antagonist, could block the oral SCC-promoting nerve-cancer crosstalk.

In oral SCC, CGRP-driven immunosuppression has correlated with unfavorable patient prognosis (283). Of note, Zhang et al. demonstrated a positive correlation between elevated α-CGRP concentration in blood plasma and the presence of PNI and lymph node metastases among patients with oral cancer (31, 284). α-CGRP levels were also higher in cancer tissue with evidence of PNI and lymph node metastases, in comparison to surrounding non-cancerous tissues and cancer tissue with the absence of PNI (284). In the oral SCC murine model, cancer cells induce CGRP receptor (CGRPR) overexpression in trigeminal ganglion neurons, which results in mechanical cancer allodynia (276, 285). Additionally, receptor activity-modifying protein 1 (RAMP1), a key component of CGRP receptors, overexpressed in oral cancer cells, may potentially be associated with the promotion of oral cancer (285). Importantly, the addition of olcegepant, the CGRPR antagonist, suppressed the degree of nociception in mice (285). Various cells within the oral TME express the CGRPR, among which fibroblasts and immune cells constituted the most frequent CGRPR-positive inhabitants of the tumor milieu (285). It is also possible that the occurrence of α-CGRP-positive nerves within the TME is associated with pain sensation (285). In addition to these findings, oral cancer cells make use of the TME to thrive in nutrient-poor environments, as nutrient-poor conditions drive cancer cell-derived NGF secretion, which promotes the release of CGRP from nociceptive nerves, eventually stimulating cytoprotective autophagy in cancer cells (256).

Recent studies also highlight that specific neuronal changes correlate with patient-reported and functional outcomes in surgically treated HPV-associated oropharyngeal SCC (OPSCC) patients (286). Enrichment of adrenergic (TH+), CGRP+ sensory, and immature cholinergic (choline acetyltransferase - ChAT+, doublecortin - DCX+) nerves, was associated with poorer swallowing outcomes in OPSCC survivors (286). Murine models further confirmed that CGRP+ and immature cholinergic nerves are linked to radiation-induced dysphagia (286).

#### 5.3.3 Substance P

The importance of substance P and its neurokinin-1 receptor (NK-1R) axis has recently gained interest for its possible role in carcinogenesis (287, 288). A recent meta-analysis demonstrated that SP and NK-1R are upregulated in pre-malignant and cancerous HN lesions compared to benign lesions (288). Therefore, SP/NK-1R axis appears to be an early event in human HN oncogenesis (288). Across HNCs, diverse expression patterns of SP/NK-1R were detected. It was demonstrated that SP/NK-1R is upregulated in laryngeal carcinomas and oral SCCs, whereas this axis shows no overexpression in malignant tumors of the salivary gland (288). As SP/NK-1R overexpression correlates with Ki-67 (a cellular marker for proliferation) upregulation, uncontrolled proliferation may be the SP/NK-1R axis contribution to carcinogenesis in HNCs (288). Especially in HN SCCs, SP/NK-1R activation enhances cell proliferation and migration ability (289). In addition, via interactions with factors such as NF-kB, ERK 1/2, and HIF1-α, SP/NK-1R axis activation leads to transformation to the more mobile and invasive phenotype of cancer cells, resulting in cancer invasion and metastasis in numerous cancers, including HNCs (290–292).

#### 5.3.4 Conclusion and future directions

**FIGURE 5:**
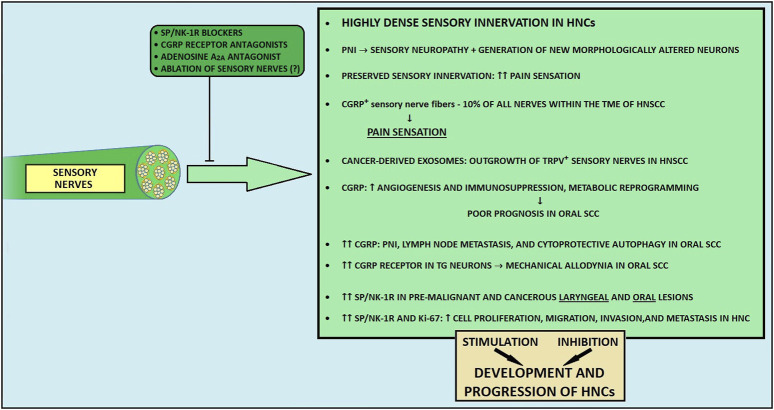
The influence of sensory nerves on development and progression of head and neck cancers. CGRP, calcitonin gene-related peptide; HNC, head and neck cancer; HNSCC, head and neck squamous cell carcinoma; NE, norepinephrine; NK-1R, neurokinin-1 receptor; PNI, perineural invasion; SP, substance P; TG, trigeminal ganglion; TRPV1, transient receptor potential vanilloid 1.

In essence, sensory nerves contribute to the development of HNCs, and more importantly, their fatal relationship with cancer cells results in increased pain sensation, leading to a decline in quality of life among cancer patients (23, 32, 293, 294) (Figure 5). In some HNC patients, adequate pain control cannot be achieved with conventional analgesic therapies (295–299). Promisingly, targeting both A_2A_R-CGRP circuit and SP/NK-1R-related pathway may help improve pain management in patients with HNCs. Moreover, eliminating CGRP transmission could enhance anti-tumor immunity. Finally, to effectively address swallowing impairments in cancer survivors and enhance anti-tumor immunity, there is a significant potential for early therapeutic interventions and the application of neurology-related drugs, such as CGRP blockers.

## 6 Discussion

In recent years, TME has gained tremendous attention in cancer research. Various nerve fibers, including sympathetic, parasympathetic, and sensory nerves, infiltrate the TME and are considered its essential components. Interaction between cancer cells and neurons manifesting as PNI, axonogenesis, neurogenesis, and nerve reprogramming are beginning to emerge as key contributors at various stages of tumorigenesis. Abundant evidence indicates that cancer cells have the ability to hijack locoregional nerves. As a result, the primary tumor receives many factors needed for local development and formation of distant metastases. Nerve-cancer crosstalk is complex and not fully understood. Therefore, future research should focus on elucidating the molecular mechanisms underlying the role of neural components in carcinogenesis. Hopefully, with a better understanding of nerve-cancer interactions, novel possible drug targets will be identified, thus leading to the development of novel clinically relevant targeted therapies.

Novel data indicate that cancers harbor higher electrical activity as compared to benign or normal tissues (289). Furthermore, both sympathetic and sensory nerves presumably contribute to increased intratumoral electrical activity, exhibiting sex-specific patterns in neural activity within tumors (289, 300, 301). In HN SCCs, tumor-infiltrating nerves likely establish functional connections with tumor cells, suggesting the possible formation of synapses or synapse-like structures within HNCs (289). To date, functional synapses between cancer cells and neurons have been described in gliomas, breast-to-brain metastasis, and small cell lung cancers (302). It is not known, however, whether functional *bona fide* synapses between neurons and other malignancies are formed.

Another relevant aspect in cancer neuroscience is the definition of PNI. As multiple studies have demonstrated that reporting PNI in a dichotomous fashion (present or absent) might not be accurate enough to divide cancer patients into distinct prognostic subgroups (104, 105, 109), further consensus should be reached regarding the description of PNI to provide more clinically relevant information. Several histologic parameters of PNI, including the focality of nerve invasion and depth of the nerve infiltration are reliable predictors of patients’ survival (44, 105–107). Therefore, more appropriate descriptions of PNI could potentially support the decision-making process for optimal cancer treatment. Advanced technologies like radiomics and AI-based approaches hold promise for reducing variability in PNI assessment, improving detection rates, and accelerating diagnosis, particularly in HNCs (86–89). Additionally, the potential integration of PNI into the TNM classification system or the development of a specific PNI staging system could further refine risk stratification and treatment planning (303, 304).

Undoubtedly the peripheral nervous system is acknowledged as a contributor to the development and progression of HNCs, due to the abundant innervation in the HN region (51, 52, 198, 202, 216, 253). Although the vast majority of studies reported that the sympathetic nervous system exerts cancer-promoting effects in HNCs (39, 51, 52, 198, 202), novel findings suggest a potent cancer-protective role of sympathetic nerves in these cancers (203, 204). A potential explanation for the double-edged sword role of sympathetic nerves in the carcinogenesis of HNCs might be the existence of several molecularly diverse subtypes of sympathetic neurons within the TME, involving different signaling pathways (206–209). Eventually, using more selective drugs inhibiting sympathetic activity could lead to better outcomes for HNC patients in the future.

On the other hand, parasympathetic nerves are likely to promote HN tumorigenesis (52, 209, 227, 253). Recent studies highlighted the role of muscarinic and nicotinic AChRs in the development and progression of HNCs. For instance, α7 nAChR may be a promising predictive biomarker of response to the frequently used chemotherapeutic drugs in HNCs (227, 236–239). Also, pharmacological modulation of the α7 nAChR-mediated signaling could be beneficial among patients with HNCs. Therefore, future research should focus on the development of novel, highly specific nAChRs inhibitors, as their clinical use could improve the therapeutic effects of currently available anti-cancer agents used in the treatment of HNCs. In addition, further studies should explore the clinical significance and utility of ACh-degrading enzymes in patients with HN malignancies.

The risk of developing HNCs is strongly associated with smoking (23, 25, 26). As tobacco-derived nitrosamines and nicotine can contribute to cancer development due to the activation of nAChRs (216, 225), quitting smoking should be encouraged whenever feasible. Given that smoking cessation after diagnosis improves survival in patients with HNC, lung cancer and breast cancer, interventions to support smoking cessation are of paramount importance. (305–309).

Patients with HN malignancies frequently experience high levels of pain, a symptom related to the spread of the primary tumor or to its multimodal treatment (23, 32, 294, 295). The involvement of sensory nerve fibers in pain sensation has recently gained the attention of researchers (21, 117, 271, 283, 285, 287–289). In some HNC patients, pain cannot be controlled using conventional pain-relief drugs (296–299). The association of cancer-related pain with gender is still unresolved (309), with some data suggesting that women with HNCs experience pain more frequently than men (309). Novel findings obtained from the mouse model indicate that females may have more sensory neurons innervating the tongue than males, with different percentages of TRPV1-positive lingual neurons between the two sexes (310). Therefore, sex-dependent differences should be taken into account to optimize cancer pain management. It is also worth exploring whether certain groups of patients with HNCs (for instance, HPV-positive vs. HPV-negative, male vs. female) require different pain management.

HPV status plays a critical role in the innervation and progression of HNCs. While HPV-negative tumors are typically more densely innervated, partly due to TP53 mutations, which may contribute to their more aggressive nature and increased pain, HPV-positive tumors, although less densely innervated, promote tumor growth through mechanisms such as *CCND1* amplification and the release of exosomal oncogenes like E6 and E7 (21, 31, 123, 124, 126, 275). Understanding these distinct patterns of innervation and their underlying molecular mechanisms could lead to more targeted treatments and improved outcomes for HNC patients based on their HPV status.

Recent research highlighted the role of cancer-associated nerves in immune regulation (118, 273). It has been demonstrated that cancer-induced nerve damage is associated not only with consequent inflammation but also with resistance to immune checkpoint inhibitors in cutaneous SCC (120). Furthermore, both denervation and blockade of pro-inflammatory IL-6 significantly enhanced anti-PD-1 efficacy in the murine model (120). In HN SCCs, response rates to immune checkpoint blockade therapies are typically low (10%–20%) (120, 121, 191). Therefore, it is of great significance to clarify whether the addition of targeted anti-inflammatory agents to treatment with immune checkpoint inhibitors can boost the response to immunotherapy. It should be also investigated if surgical or pharmacological denervation could represent a novel approach to improve the response to immune therapies in various neurotrophic cancers, such as prostate and pancreatic malignancies. Translating denervation approaches in cancer patients, although promising from a theoretical standpoint, poses significant challenges. Especially in malignancies of head and neck region (such as tongue or oropharyngeal cancers), surgical denervation can be associated with serious side effects (311). Therefore, more selective and innovative approaches (surgical, pharmacological, or genetic) are needed to minimize side-effects of denervation while maintaining its efficacy.

In conclusion, the interplay between cancer and the nervous system is complex and multidimensional, engaging numerous factors. Although our understanding of cancer has significantly improved over time, it is only recently that the role of nerves in carcinogenesis has been given notable attention. Further studies are needed to elucidate the underlying molecular mechanisms of cancer-related nerve phenomena. Eventually, novel insights into the nerve-cancer crosstalk might lead to a potential application of nerve-targeting strategies in oncology.

## Glossary

ACC: adenoid cystic carcinoma
ACh: acetylcholine
AChE: acetylcholinesterase
BChE: butyrylcholinesterase
BDNF: brain-derived neurotrophic factor
CGRP: calcitonin gene-related peptide
CHAT: choline acetyltransferase
CGRPR: calcitonin gene-related peptide receptor
CN: cranial nerve
CSCs: cancer stem cells
DCX: doublecortin
DFS: disease-free survival
DSS: disease-specific survival
DRG: dorsal root ganglia
ECM: extracellular matrix
EGFR: epidermal growth factor receptor
EMT: epithelial-to-mesenchymal transition
ENI: endoneural invasion
Gal: galanin
GalR: galanin receptor
GDNF: glial cell-derived neurotrophic factor
GFAP: glial fibrillary acidic protein
GFRα1: GDNF Family Receptor-α1
HNCs: head and neck cancers
HPV: human papillomavirus
Ig-CAM: immunoglobulin-like cell-adhesion molecules
IL-6: interleukin 6
mAChR: muscarinic acetylcholine receptor
MET: mesenchymal-to-epithelial transition
MMPs: metalloproteinases
MRI: magnetic resonance imaging
nAChR: nicotinic acetylcholine receptor
NCAM1: neural cell adhesion molecule 1
NE: norepinephrine
NGF: nerve growth factor
NK-1R: neurokinin-1 receptor
NNK: nicotine-derived nitrosamine ketone
NPY: neuropeptide Y
NT: neurotrophin
OS: overall survival
PNI: perineural invasion
PNS: perineural spread
RET: rearranged during transfection
SCC: squamous cell carcinoma
SCs: Schwann cells
SP: substance P
TCGA: The Cancer Genome Atlas
TH: tyrosine hydroxylase
TME: tumor microenvironment
TrkA: tropomyosin receptor kinase A
TrkB: tropomyosin receptor kinase B
TRPA1: transient receptor potential ankyrin 1
TRPV1: transient receptor potential vanilloid 1
WPNI: worst pattern of PNI
